# Paroxysmal Positional Nystagmus in Acoustic Neuroma Patients

**DOI:** 10.3390/audiolres13020026

**Published:** 2023-04-13

**Authors:** Francesco Comacchio, Paola Magnavita, Barbara Bellemo

**Affiliations:** 1 Otolaryngology Unit and Vertigo Regional Specialized Center, Sant’Antonio Hospital, Via J. Facciolati 71, 35127 Padova, Italy; 2 Department of Surgery, Padova University Hospital, Via Giustiniani 2, 35128 Padova, Italy

**Keywords:** paroxysmal positional nystagmus, benign paroxysmal positional vertigo, acoustic neuroma, light cupula, heavy cupula, videonystagmography

## Abstract

The association between acoustic neuroma and positional vertigo with paroxysmal positional nystagmus is relatively rare, but, when present, it certainly represents a challenge for the otoneurologist. There are few reports in the literature on this particular issue, and some questions are still unanswered, particularly regarding the characteristics of positional nystagmus that may distinguish between a true benign paroxysmal vertigo and a positional nystagmus associated with the tumor. We present the videonystagmographic patterns of seven patients with acoustic tumor who had paroxysmal positional nystagmus and analyzed its features. A concomitant true benign paroxysmal positional vertigo may be present during the follow-up of a non-treated patient, as the paroxysmal positional vertigo may be the first symptom of the tumor, and it may show characteristics that are very similar to a posterior semicircular canal canalolithiasis or a horizontal canal “heavy or light cupula”. The possible mechanisms are discussed.

## 1. Introduction

Paroxysmal positional nystagmus (PPN) is a common finding in patients with vertigo and can occur in typical and atypical forms. In its typical form, PPN is usually caused by benign paroxysmal positional vertigo (BPPV), a common cause of peripheral vertigo. Atypical PPN could be caused by conditions which are not benign such as central nervous system pathologies. The association between acoustic neuroma (AN) and PPN is relatively rare [1], but when present, it can represent a challenge for the otoneurologist. AN patients may sometimes present paroxysmal positional vertigo as their first symptom, or episodes of BPPV may occur during the observation period of the tumor. The characteristics of PPN may be very useful in the differential diagnosis between true BPPV and atypical forms with pseudo-benign paroxysmal positional vertigo or “malignant” paroxysmal vertigo [2,3] due to ischemic or neoplastic lesions in the central vestibular pathways. The typical nystagmus of BPPV includes transient nystagmus with latency, crescendo and decrescendo patterns, and fatiguing upon provocation, and the condition usually resolves after liberatory procedures. When this pattern of nystagmus is absent, especially when there is no latency and no fatigability, and the vertigo is resistant to liberatory maneuvers, a central pathology is suspected and a neuroradiological evaluation with brain MRI is required. Here, we discuss the findings of our case series of AN patients with PPN.

## 2. Materials and Methods

All AN patients that presented with PPN were considered among 5100 patients seen at the Regional Specialized Center of Vertigo of University Hospital of Padova during the last seven years. All patients presented with a well-documented AN at MRI. They underwent to computerized videonystagmography before possible surgical therapy, and the presence of spontaneous and positional nystagmus was recorded using an ICS computerized videonystagmographic system. The following PPN characteristics were studied: the presence of rotatory vertical up- or down-beating or horizontal direction-changing positional nystagmus, latency, a crescendo–decrescendo pattern, exhaustibility, and their response to liberatory maneuvres. The videonystagmographic study was completed by analyzing saccadic and smooth pursuit ocular movements and bithermal calorics.

## 3. Results

Seven patients were found in our database presenting AN and PPN. The global results of the case series are reported in Table 1.

**Figure 1:**
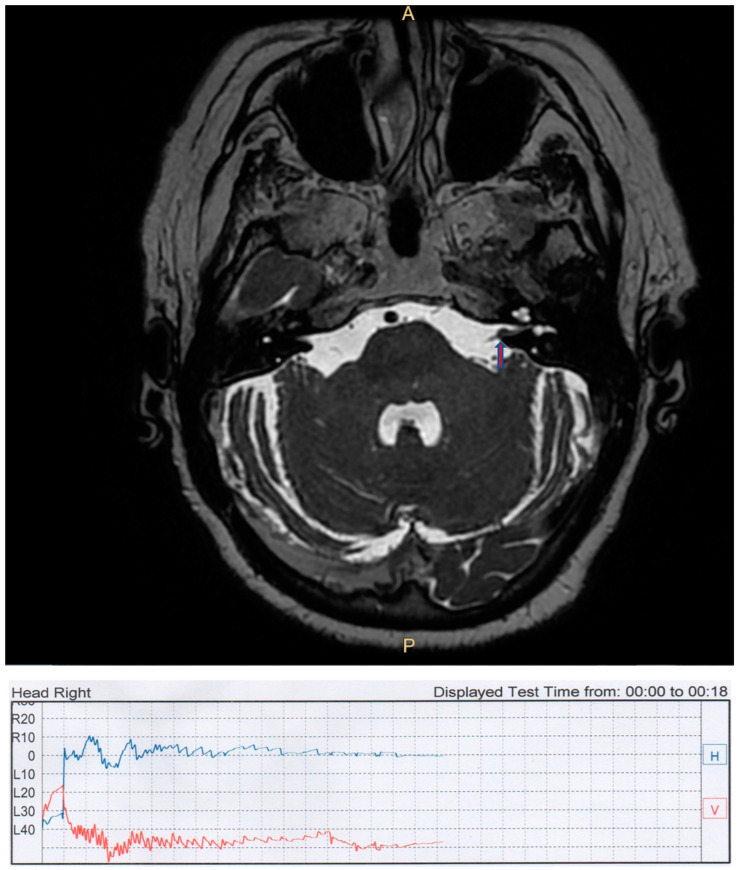
Case TL. (**Top**): brain axial T2 cube MRI with a left-side intrameatal AN (arrow). (**Bottom**): videonistagmographic recording of contralateral PSC BPPV.

All patients were female, with a mean age of 67.4 years (range of 49–81). In five patients, positional vertigo was the first symptom of the tumor, and in two cases, PPN occurred during the follow-up period. These last two patients (TL and DF) were both affected by a right intrameatal AN and both presented with classic posterior semicircular canal (PSC) BPPV with the typical rotatory vertical up-beating nystagmus with latency, a crescendo–decrescendo pattern, and fatigability (Figure 1).

**Figure 2:**
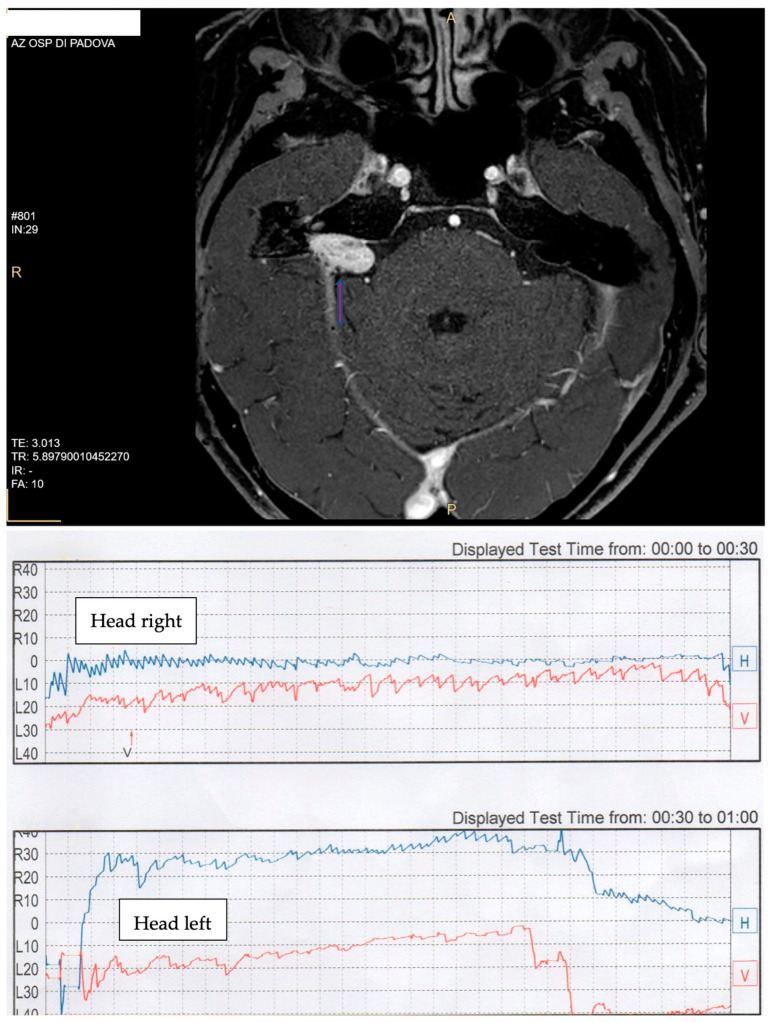
Case CC. (**Top**): AN in the right CPA (arrow). (**Bottom**): videonistagmographic recording of Pagnini–McClure positional test. A geotropic direction-changing horizontal nystagmus was present, with a crescendo–decrescendo pattern, short latency, and a vertical downbeat component in the right side (video).

**Figure 3:**
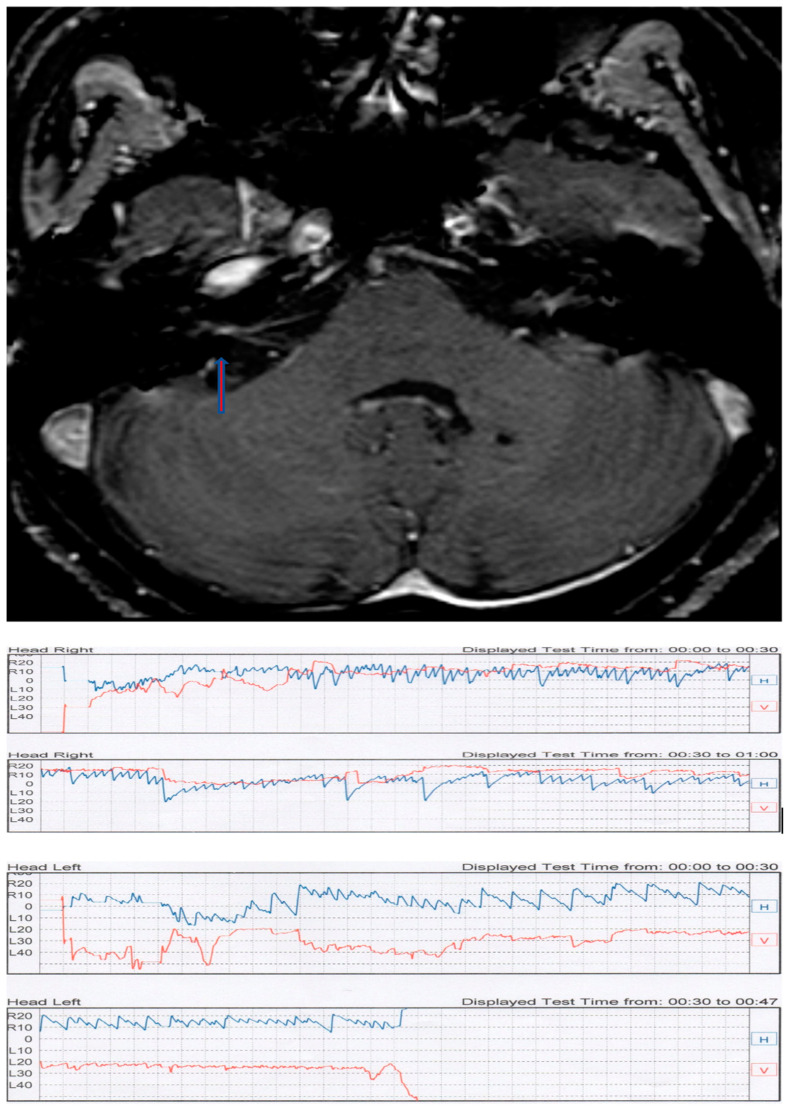
Case BP. (**Top**): intrameatal AN of the right side (arrow). (**Bottom**): videonystagmographic recording of the Pagnini–McClure positional test. An apogeotropic horizontal direction-changing positional nystagmus is present with a paroxysmal component in the affected side.

TL presented with PSC BPPV in the contralateral side of the tumor. In both cases, a liberatory maneuver, according to Semont, was successful and completely solved BPPV. In two patients (CC and BP), a direction-changing positional horizontal nystagmus was found. CC (Figure 2) presented with geotropic PPN in the right side with no latency but with fatigability and a crescendo–decrescendo pattern. When the horizontal component seemed to disappear, a persistent vertical downbeat component appeared on the right side as well (see video in Supplementary Materials). The paroxysmal component which was evident in the right side was less present on the left side. These findings suggest a central pathology. An MRI scan of the brain revealed a right AN protruding in the pontocerebellar angle (PCA). In this case, the tumor was recurrent and had been surgically treated ten years earlier. In this case, a revision surgery was performed. BP demonstrated apogeotropic direction-changing PPN with a crescendo–decrescendo pattern, a long duration (more than 1 min), and a latency less than a second (Figure 3).

**Figure 4:**
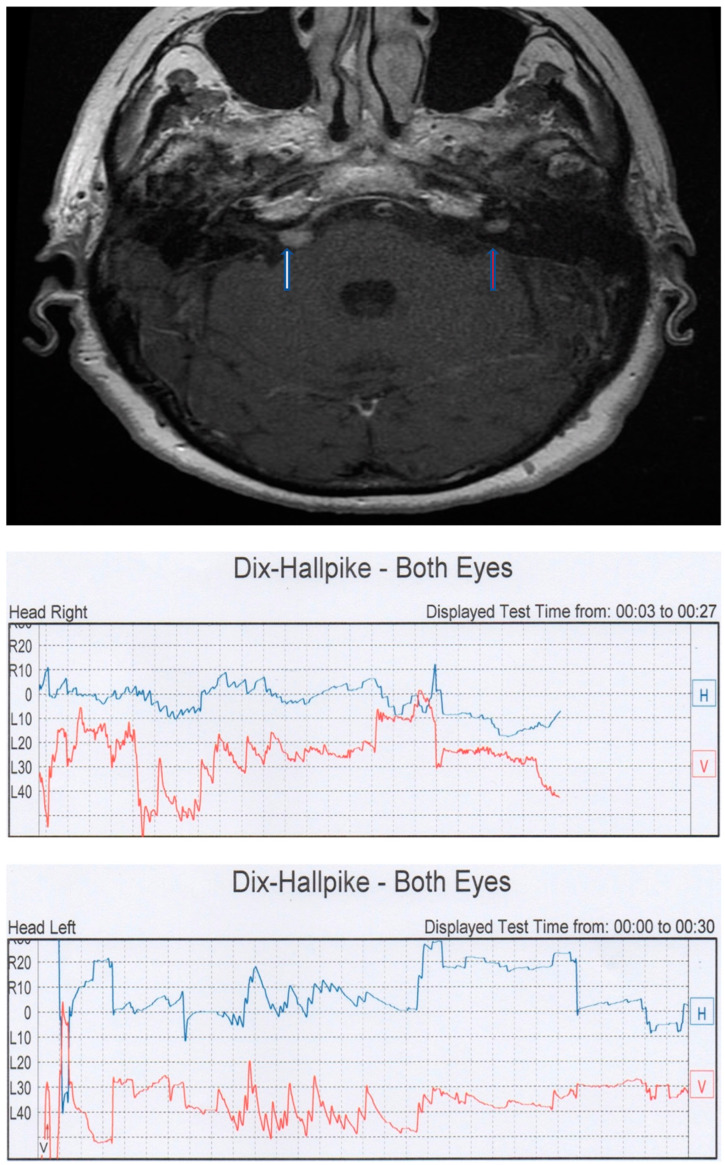
Case CT. (**Top**): right CPA meningioma (white arrow) and left intrameatal AN (red arrow) (courtesy of E. Zanoletti). (**Bottom**): videonystagmographic recording of the Dix–Hallpike test showing a bilateral classic PSC BPPV picture.

Moreover, in this case, the paroxysmal component was more evident on the pathological side. A brain MRI documented a small intrameatal AN on the right side. Cases BM and VN were strikingly similar. Both presented an intrameatal AN; in the case of VN, a falx meningioma was also present in the context of a neurofibromatosis type 2 syndrome (NF2). In these cases, PPN presented characteristics similar to classic PSC BPPV, but with very short latency, poor fatigability, and a lack of resolution after liberatory maneuvers. BM presented with the same patterns of nystagmus at three different controls during a six-year follow up. More challenging is the last case, CT, a NF2 patient affected by a meningioma in the PCA on the right side and an intrameatal AN of 6 mm on the left side. The patient presented with bilateral classic PSC BPPV with latency, a crescendo–decrescendo pattern, and with exhaustible nystagmus (Figure 4). The first symptom that led to the diagnosis of NF2 was positional vertigo. The patient presented with brief periods of remission after liberatory maneuvers, but with a high frequency of recurrences.

## 4. Discussion

The association between AN and PPN can represent a challenge for otoneurologists when differentiating between classic BPPV or PPN not related to an otolithic detachment. In a review published by Riga et al. [4], the most commonly recognized conditions associated with secondary BPPV are head trauma, vestibular neuritis, Ménière’s disease, and postsurgical conditions. Other lesions that affect the inner ear and have been implicated in the pathogenesis of secondary BPPV are sudden sensorineural hearing loss and migraine. Secondary BPPV seems to be under-diagnosed because dizziness may be atypical and attributed to the primary inner ear pathology. AN is usually not considered as a possible cause of secondary BPPV. The clinical differences between idiopathic and secondary BPPV demonstrated by a large case series are of limited clinical value. Most studies agree that secondary BPPV is more difficult to treat than idiopathic and that patients often require longer time intervals before becoming free of any symptoms. The clinical manifestation of an additional inner ear or more central lesion may be a possible explanation. Our data confirm that PPN may be the first sign of a suspected lesion that is more central or that classic BPPV may occur during the follow-up of non-treated AN. In this case, it may be successfully treated with liberatory maneuvers. The diagnosis is straightforward when present on the contralateral side of a well-known AN, but when classic BPPV is ipsilateral to the tumor, the question arises of whether AN might be a predisposing factor to BPPV. Dunniway and Welling [5] described a case of classic PSC BPPV that presented with sudden hearing loss in the same ear after a few months, and AN was diagnosed. Karatayli-Ozgursoy et al. [6] described an analogous case of AN detected in a patient who suffered from BPPV episodes for many years. In a study by Sahyouni et al. [7], 22% of AN patients with other vestibular pathologies also had BPPV. Patients with episodic vertigo should be distinguished from those with a chronic-tumor-induced imbalance. Our case (CT) with frequent bilateral BPPV recurrences seems to confirm that there may be a link between vestibular schwannoma and BPPV; however, possible causes remain unknown. A more difficult issue is PPN mimicking PSC BPPV, but with more subtle differences in the patterns presented. Two patients (BM and VN) presented with a crescendo–decrescendo pattern, if not as evidently as in classic PSC BPPV. In one of our previous reports [8], a lack of a crescendo–decrescendo pattern in PPN was associated with central cerebellar pathology. However, in the past, the presence of a crescendo–decrescendo pattern was also associated with a central lesion [9], as well as PPN with other peripheral characteristics such as fatigability and latency [10]; Sakata considered these patients to be affected by pseudo-BPPV and described the presence of a cerebellar vermis lesion in all of his series. So, it may sometimes be challenging to distinguish a peripheral pattern from a central pattern based on these features. Our two patients, however, presented with both a very short latency as opposed to secondary BPPV, and a lack of response to liberatory maneuvers was reported as their main characteristic. The failure of repositioning maneuvers was also described by Hernandez-Montero et al. [11] and by Noh et al. [12] in a case of posterior cerebellar fossa meningioma and cerebellar hemangioblastoma, respectively, mimicking BPPV. Moreover, the absence of symptomatology, often described as a differential feature between BPPV and other central pathologies [13], was not present in our series. All the patients with PPN, which is similar to PSC BPPV, complained of some positional symptoms. The findings of direction-changing horizontal PPN in AN patients are also challenging. One of our two patients with horizontal direction-changing PPN presented a geotropic form. A crescendo–decrescendo pattern was recorded in this patient, but the difference with classic horizontal canal (HC) BPPV was the lack of latency when the head was turned on both sides. This case was more similar to the pathologic findings encountered in patients affected by “light cupula”, but the paroxysmal characteristic is usually not evident in “light cupula”. Hiruma [14] described a case of intrameatal AN that presented with classic geotropic direction-changing PPN with the characteristics of a light cupula, no latency, a neutral point, and no decadency of slow-phase angular velocity (SPV). The author hypothesized that AN located in the internal auditory meatus at the early stage could affect peripheral endo-organs such as the lateral semi-circular canal. Our case did not present light cupula features because of the presence of a crescendo–decrescendo pattern of SPV and the lack of a neutral point, but as a distinctive sign, it also presented a vertical downbeat component on the affected side. Furthermore, if the geotropic direction-changing PPN has been attributed to a floccular lesion [15,16], our patient presented with a tumor protruding in the PCA and previous surgery results in that area, so a clear central component on the PPN is possible. Carmona et al. [17] also described another case of AN with short-latency geotropic PPN and no response to liberatory maneuvers. A case similar to our other case, with apogeotropic-type horizontal direction-changing PPN, is described in the literature. Taylor et al. [18] reported an AN patient who presented with positional nystagmus mimicking HC cupulolithiasis. The only differential feature was the stronger apogeotropic positional nystagmus on the affected side. Our case shares this finding, and this observation may be the unique differentiation between these cases and a classic HC cupulolithiasis. With the exception of three patients, Choi et al. [19] observed the same characteristic of stronger ipsilesional nystagmus in a series of patients with apogeotropic direction-changing positional nystagmus caused by central lesions. Furthermore, when compared to the minor differences in the central positional group, the authors discovered a greater asymmetry of nystagmus SPV for the affected side versus the healthy side. Johkura et al. [20] observed that the latency of PPN was always greater than 2 s in BPPV patients affected by HC cupulolithiasis, as compared to patients suffering from cerebellar lesions, in which the latency was inferior to 1 s, and the amplitude of SPV was smaller than that of BPPV. Our patients also seem to follow this rule. In summary, some key points suggesting a retro-labyrinthine pathology are:

- the lack of any resolution after liberatory maneuvers;
- the lack of a crescendo–decrescendo pattern of PPN;
- the presence of vertical component in HC PPN, especially in the affected side;
- latency <2 s in the case of direction-changing horizontal PPN;
- in AN cases mimicking a HC cupulolithiasis, the affected side has stronger nystagmus.

Due to the presence of only subtle differences between cupulolithiasis and AN patients with apogeotropic PPN, a diligent follow-up in all patients diagnosed with cupulolithiasis is suggested, particularly for those who were not responsive to liberatory maneuvers, which are often not immediately resolutive in these patients. However, the mechanism by which a small intrameatal acoustic neuroma might generate central-type direction-changing positional nystagmus remains an unsolved issue. Possible attempts to explain these findings are the presence of an atypical form of cupulolithiasis that has not been responsive for years, or the presence of a previously undetected central dysfunction at the level of the velocity storage mechanism (VSM) or the cerebellar vermis that could coexist in some patients; in these conditions, the pathological input from the peripheral vestibular system may not adequately compensate in the center. Peripheral lesions, such as Meniere’s disease attacks or BPPV, which are capable of generating central types of nystagmus, such as periodic alternating nystagmus or windmill nystagmus, both involving the VSM, are described in the literature [21,22,23].

## 5. Conclusions

In conclusion, our series demonstrates that AN and BPPV may be concurrently present during the follow-up period of untreated patients and that symptoms may be resolved with physical treatment. A videonystagmographic image resembling both PSC BPPV and HC BPPV, in geotropic and apogeotrophic forms, may be the first signs of AN, but a careful analysis of nystagmus features usually reveals some differences from classic canalolithiasis or cupulolithiasis, allowing the correct diagnosis to be reached.

## Figures and Tables

**Table 1:** Global characteristics of the patients. * Only the side with AN is reported. CT also has contralateral PSC PPN with a PCA meningioma (see Figure 4).

Patient	Sex	Age	Tumor Characteristics	Tumor Side *	PPN	Time of Onset	Liberatory Maneuvers
TL	female	69	intrameatal (0.5 cm)	left	left PSC BPPV	follow-up	resolutive
DF	female	49	intrameatal (0.7 cm)	right	left PSC BPPV	follow-up	resolutive
CC	female	69	protruding CPA	right	geotropic direction-changing PPN	first sign	not responsive
BP	female	54	intrameatal (0.54 cm)	right	apogeotropic direction-changing PPN	first sign	not responsive
BM	female	81	intrameatal (0.3 cm)	left	left PSC PPN	first sign	not responsive
VN	female	75	intrameatal (0.5 cm) with NF2 multiple meningiomas	left	left PSC PPN	first sign	not responsive
CT	female	75	intrameatal (0.7 cm) NF2 (CPA right meningioma)	left	left PSC BPPV	first sign	responsive with frequent recurrences

## Data Availability

The data presented in this study are available upon request from the corresponding author. The institutional policy is that a formal data-sharing agreement is required to share the data with outside researchers.

## Supplementary Materials

The following supporting information can be downloaded at: https://www.mdpi.com/article/10.3390/audiolres13020026/s1.
